# Supplementary material for: Nutritional and Technological Optimization of Wheat-Chickpea- Milk Powder Composite Flour and Its Impact on Rheological and Sensorial Properties of Leavened Flat Bread
Source: Foods. 2021 Aug 10;10(8):1843. doi: 10.3390/foods10081843 (PMC8391890; doi:10.3390/foods10081843)
Supplement: Supplementary file 1 [file foods-10-01843-s001.zip › foods-1310434-supplementary.pdf]

Supplementary material

Table S 1 Graphical and numerical optimization of composite flour

	**Graphical optimization**	**Numerical optimization**
**Proteins(g/100gDM)**	15,1969	15,1971
**Lipids(g/100gDM)**	6,83004	6,83052
**Carbohydrats(g/100gDM)**	64,5233	64,5229
**Fe(ppm)**	57,6145	57,6143
**Zn(ppm)**	45,4751	45,4758
**P(ppm)**	4575,32	4418,82
**Polyphenols(µgGAE/gDM)**	563,016	563,012
**Scavenging activity(%)**	17,0955	17,0957
**Yellow index**	19,6008	19,6
**Zeleny number(ml)**	10,0983	10,0783

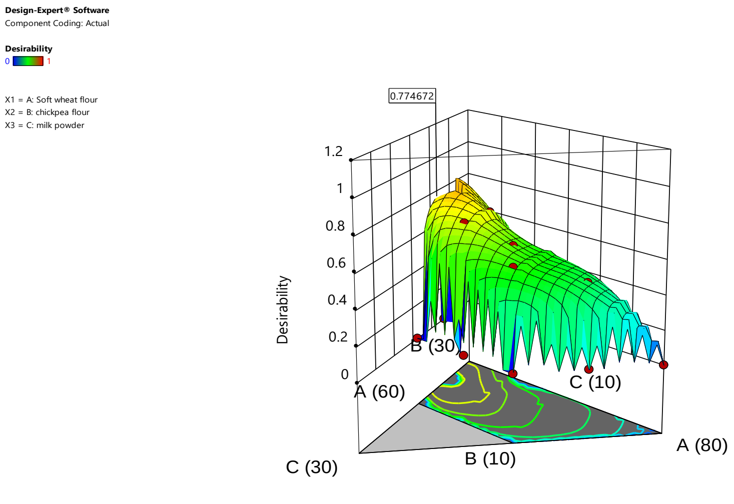

Figure S1: Response surface mixture optimization of composite flour

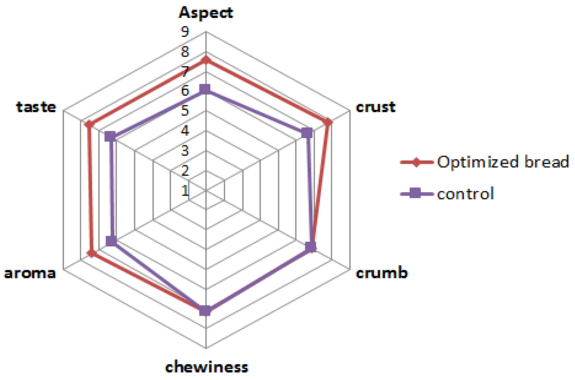

Figure S2: Average acceptance for optimized and control breads

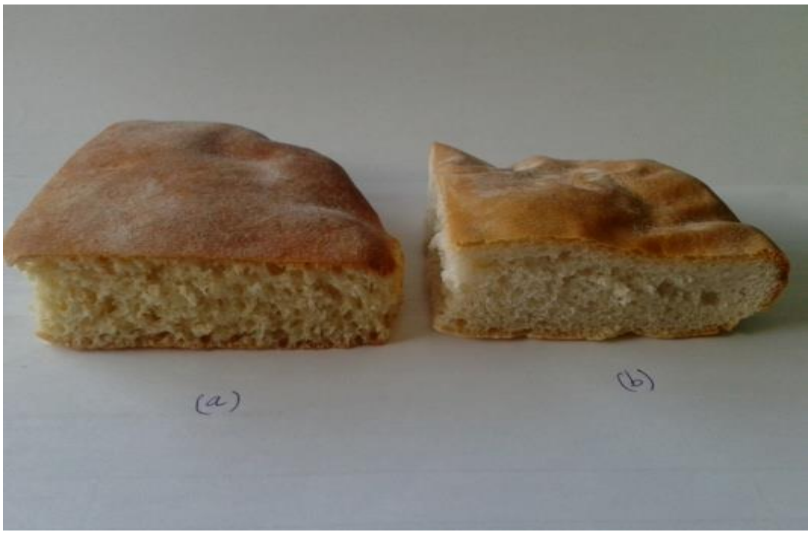

Photo S1: Formulated breads: from left a)-optimized bread, b)-bread wheat bread (control)
